# Acromial Stress Fractures in Reverse Total Shoulder Arthroplasty: Diagnosis, Prevention, and Treatment

**DOI:** 10.1007/s12178-026-10054-7

**Published:** 2026-08-18

**Authors:** Lauren J. Hsue, Mathangi Sridharan, Kushagra Tewari, Nicole J. Newman-Hung, Nicole M. Truong, Edward C. Cheung

**Affiliations:** 1 Department of Orthopaedic Surgery, University of California Los Angeles, Los Angeles, CA USA; 2 David Geffen School of Medicine, Los Angeles, CA USA; 3 1225 15th Street Suite 2100, Santa Monica, CA 90404 USA

## Abstract

**Purpose of Review:**

This review critically evaluates the literature on acromial stress fractures (ASFs) following reverse total shoulder arthroplasty (rTSA), with a focus on risk factors, prevention, and treatment. By synthesizing contemporary evidence and highlighting areas of ongoing debate, this review aims to provide clinicians with an updated understanding of these complications and identify priorities for future research.

**Recent Findings:**

ASFs have an approximate incidence of 3–5% following rTSA. Preoperative risk factors include female sex, acromial morphology, rheumatologic disease, chronic systemic steroid use, and intra-articular steroid use within three months of arthroplasty. Intra-operative risk factors include implant positioning parameters such as excessive distalization, as well as prominence of posterior superior baseplate screws, and resection of the coracoacromial ligament. While nonoperative management is the first line of treatment for many ASFs, displaced fractures are commonly treated operatively. Operative intervention facilitates fracture union but does not consistently improve functional outcomes. Intranasal calcitonin is currently being studied as an adjuvant therapy. The impact of implant design features and placement decisions addressing distalization and lateralization are areas of ongoing study.

**Summary:**

ASFs remain a challenging complication of rTSA with limited treatment success, highlighting the need for better risk identification, bone health optimization, thoughtful intraoperative decision-making, and evaluation of early interventions to improve prevention and progression.

## Introduction

Reverse total shoulder arthroplasty (rTSA) has transformed surgical management of massive irreparable rotator cuff tears, rotator cuff arthropathy, proximal humerus fractures, and complex shoulder pathology. The utilization of rTSA has expanded across a diverse and aging patient population. With broader adoption, a heightened awareness of the procedure’s complications and how to avoid them is paramount.

Acromial stress fractures (ASFs) are increasingly recognized as significant rTSA complications. Occurring at rates of approximately 3–5% in modern series of primary rTSA [1, 2], ASFs have been associated with a variety of patient- and treatment-specific risk factors, including sex, bone quality, prior shoulder surgery, and excessive implant lateralization or distalization. Recently, additional attention has turned to the contribution of accelerated rehabilitation progression to ASF risk [3]. Developing strategies to avoid ASFs is essential, as outcomes of rTSA in patients with ASF are inferior to those without this complication [4]. Patients with ASFs exhibit poorer function, decreased range of motion, increased pain, and increased rates of revision rTSA [5, 6].

While recognition of risk factors and implications of ASF is improving, treatment remains difficult. Operative management with open reduction internal fixation (ORIF) has not consistently demonstrated functional benefits over nonoperative management, which remains the standard of care despite frequently failing to achieve clinically significant improvement. Fixation has its own complication profile including hardware failure, nonunion, and persistent pain [7].

Ongoing challenges in prevention and treatment highlight the need for a contemporary assessment of the literature. This review provides an updated evaluation of the diagnosis, classification, prevention, and management of ASFs following rTSA. It aims to offer a practical, evidence-informed framework for identifying at-risk patients, counseling them appropriately, and selecting management strategies when fractures occur.

## Classification

**Fig. 1:**
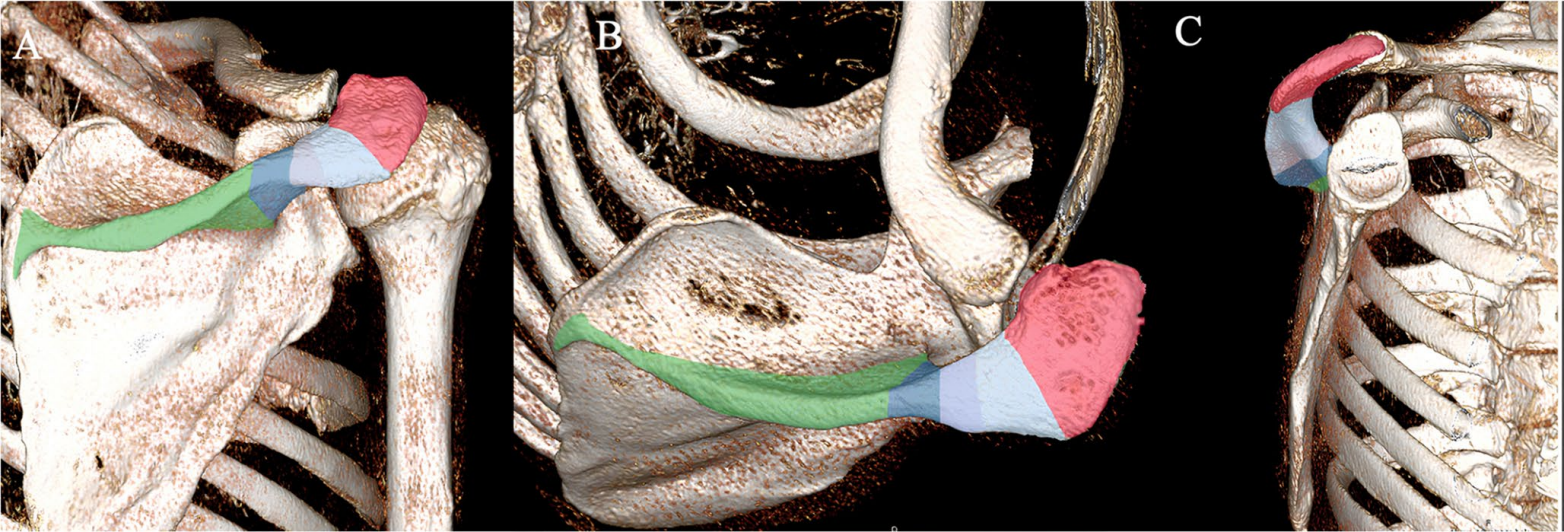
Modified Levy Classification in coronal (**A**), axial (**B**), and sagittal (**C**) 3-dimensional CT reconstructions. Zone I fractures (red) occur anterior to the posterolateral corner of the acromion. Zone II fractures (blue) occur between the posterolateral corner of the acromion and its base and are further divided into IIA (pale blue), IIB (periwinkle), and IIC (navy) subtypes. Zone III fractures (green) involve the scapular spine

The Levy classification has become the dominant system describing ASFs (Fig. 1). The primary Levy classification describes fractures based on placement within three zones of the acromion [8]. Type I fractures are located between the tip of the acromion and its posterolateral corner, involving the anterior and middle deltoid origins, and carry the best prognosis. Outcomes approach those of rTSA patients without ASF. Type II fractures are the most common and lie between the posterolateral corner of the acromion and its base. These are sub-classified into IIA fractures lateral to the glenoid face, IIB fractures at the glenoid face, and IIC fractures medial to the glenoid face but not reaching the scapular spine [9]. Type IIA fractures carry relatively favorable prognoses. IIB fractures have an intermediate prognosis with functional deficits compared to non-ASF controls [9]. The clinical relevance of the IIC fracture subtype has recently been called into question by 3D CT mapping evidence by Glener et al. suggesting there is no true fracture cluster at this location [10]. This group’s evaluation of 40 ASF patients demonstrated a roughly equal distribution of fractures across the remaining four Levy subtypes. Finally, Type III fractures are defined by scapular spine involvement. These fractures impart the worst prognosis, with increased inferior angulation of the acromion and deltoid dysfunction. Type III fractures consistently fail to achieve clinically significant improvement with nonoperative management [9].

In contrast, the Crosby classification defines ASF subtypes by relationship to the acromioclavicular (AC) joint [11]. Type I fractures are characterized by an avulsion of the anterior acromion, located at or anterior to the AC joint; as with Levy type I, these fractures are often successfully managed nonoperatively. Type II fractures occur posterior to the AC joint, and the authors hypothesized they were related to alterations in force transmission resulting from AC joint arthritis. Comparable to the Levy classification, Type III fractures involve the scapular spine, and Crosby and colleagues advocate for operative management in this scenario.

While the original Levy publication reported near-perfect reliability among raters [8], a subsequent study by Otto et al. [12] reported a more modest kappa coefficient of 0.422. Notably, Otto and colleagues provided raters with full series of plain radiographs whereas the Levy group presented raters with single images (either CT or plain radiographs) deemed most representative of the injury, which may explain the discrepancy in reported reliability. Cumulatively, these reports suggest overall good reproducibility for an anatomically based classification system that relies on obtaining appropriate imaging to make an accurate diagnosis. They highlight that plain radiographs may be misleading in the diagnosis of ASF. In contrast, there are no formal evaluations of the reproducibility of the Crosby classification in the literature.

While these systems were developed to promote a standardized method of communicating and comparing outcomes, a 2025 systematic review identified significant heterogeneity regarding use of classifications, imaging modalities, and diagnostic definitions [13]. Many series do not apply a formal classification, limiting meaningful comparisons across studies. Additionally, the predictive value of existing classifications remains limited. The Levy system has been shown to predict prognosis after nonoperative management [9]. However, newer evidence suggests that initial fracture angulation and displacement may be more important prognostic factors than fracture location [14].

### Diagnosis

#### Patient History

Classically, ASFs present as an acute increase in pain and decrease in shoulder motion in a patient previously recovering well after rTSA. Inciting trauma is identified in a minority of cases [6]. Pain is often progressive, localized to the acromion or scapular spine. Symptoms typically present months after surgery, with reported mean diagnosis at 3.5 to 10 months postoperatively [2, 15]. Pain is exacerbated by active shoulder elevation, reflecting the mechanism of increased bending stress applied to the acromion during deltoid activation. This presentation warrants a thorough physical exam and imaging workup directed toward ASF identification.

### Physical Exam

Physical exam is classically significant for acromial tenderness at the fracture site. Severe AC joint osteoarthritis with hypertrophic osteophytes may coexist with ASF, and AC joint tenderness should be assessed separately. In Levy Types IIB-III, inferior angulation of the acromion fragment may be visible or palpable.

Active forward elevation is typically more limited than passive elevation, reflecting the mechanism of bending stresses applied over the acromion with deltoid activity. Accordingly, resisted deltoid testing reproduces pain and may reveal weakness, but can be influenced by differences in pain tolerance and vary depending on the initial indication for rTSA, as an intact supraspinatus can supplement deltoid strength.

### Imaging

Plain radiographs are the initial imaging choice in an ASF workup. Standard AP, scapular Y, and axillary or Velpeau views should be obtained. These views permit assessment for suspicious lucency or displacement. However, hardware may obscure fractures, and early stress reactions or nondisplaced fractures may be radiographically occult. Many nondisplaced fractures are initially missed on x-ray and require advanced imaging for identification [13]. Os acromiale is an important imaging confounder and should be recognized not only in postoperative ASF workup but also during preoperative planning. Whether an os acromiale independently increases post-rTSA fracture risk remains unclear.

**Fig. 2:**
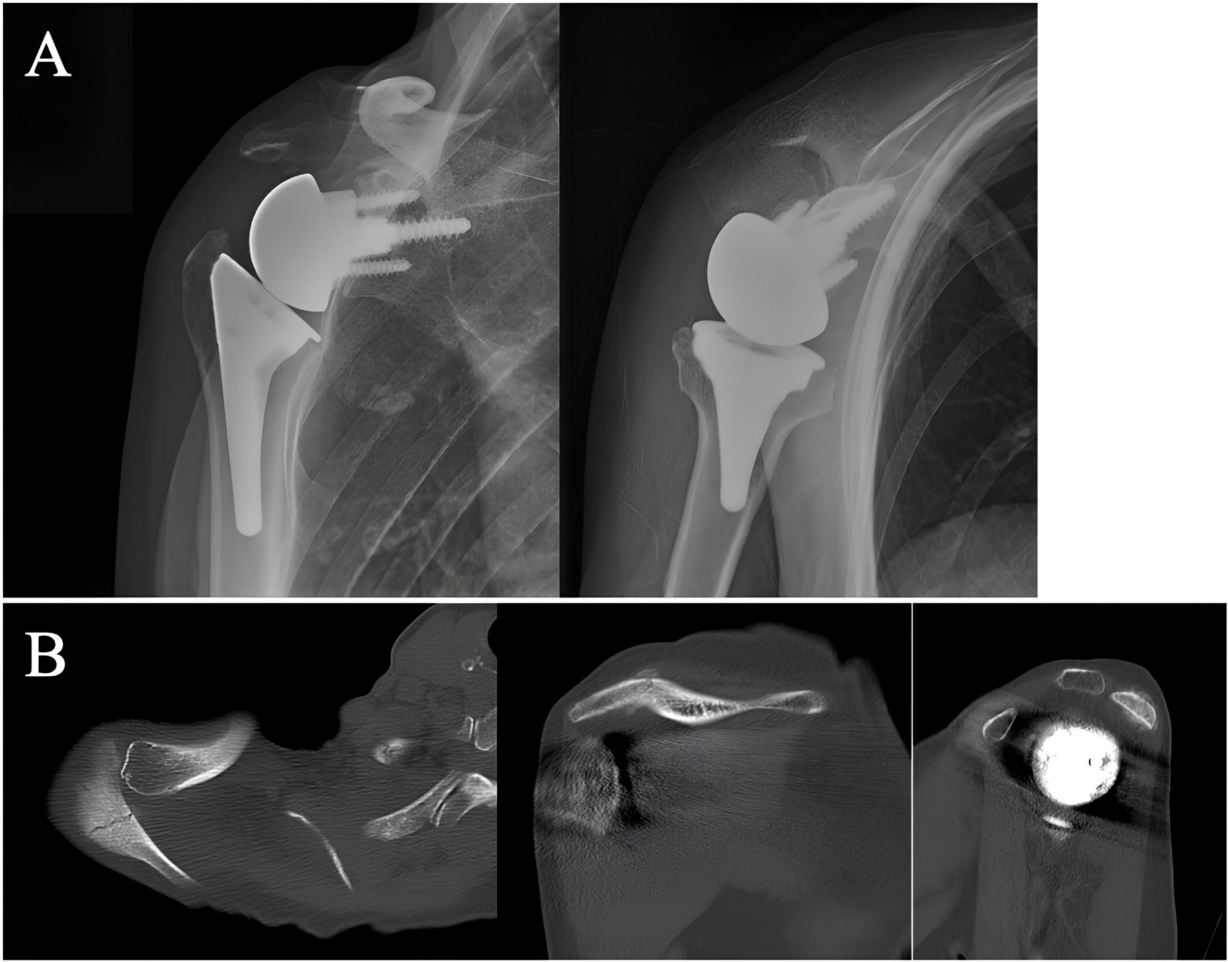
69-year-old female with new shoulder pain three months after primary rTSA, with unrevealing AP and Velpeau x-rays (**A**), found on CT to have a nondisplaced Levy zone IIA acromial stress fracture (**B**). Pain resolved with eight weeks of nonoperative management; range of motion deficits persisted but were well-tolerated

CT is the preferred confirmatory modality when clinical suspicion is high but plain radiographs are unrevealing or equivocal (Fig. 2). CT characterizes fracture location, displacement, and change in acromiohumeral distance. It also distinguishes between the sclerotic, rounded margins of an os acromiale and acute cortical disruption consistent with fracture. Metal artifact can limit image quality, and metal artifact reduction sequences (MARS) may provide supplemental information in equivocal cases. Evidence for MRI in the setting of ASF is limited. Radiological literature suggests that even with MARS, MRI one year after rTSA identifies subacromial edema in 88% of patients without correlation to differences in function. Humeral shaft edema was identified in 100% of patients, many of whom were asymptomatic, calling into question the utility of MRI in routine workup of fractures about an rTSA [16].

Acromial stress reaction (ASR) describes symptomatic post-rTSA acromial pain with bony changes on CT but without a definitive fracture line [1]. This can be conceptualized as a precursor lesion along the stress fracture continuum. Patients with ASR have functional outcomes similar to those with ASF, significantly worse than rTSA patients without either complication out to two years post-operatively [1]. Early identification of ASR may offer an opportunity for preventative intervention before fracture, although the natural history of ASR or its modifiability is not well described.

## Etiology

ASFs arise from an imbalance between increased mechanical stress, as a result of implant design and positioning, and the acromion’s capacity to resist that stress, determined by patient and anatomic factors. The biomechanical consequence of rTSA is distalization and medialization of the glenohumeral center of rotation relative to native shoulders. This produces increased deltoid lever arm, generating an increased bending moment across the acromion [17]. Recurrent loading in this manner precipitates fatigue failure, leading to a spectrum of pathology from stress reactions to stress fractures. Timely diagnosis of ASFs depends on awareness of classic presentations, high-risk patient populations, and appropriate imaging selection.

### Patient-Specific risk Factors

Investigation of patient-specific risk factors for ASF has identified female sex [6, 18], osteoporosis [3, 6, 18, 19], inflammatory arthritis [3, 6, 15, 18, 20], and older age [6, 21, 22], as summarized in Table 1. Rotator cuff pathology, including cuff tear arthropathy, has also been associated with ASF. This is thought to be related to pre-existing acromial thinning due to superior humeral head migration and acromial acetabularization [18, 22]. Similarly, prior ipsilateral shoulder surgery has been identified as a risk factor [5]: specifically, acromioplasty or prior arthroplasty have been associated with ASF, likely due to reduced acromial bone stock secondary to these procedures.

**Table 1:** Patient-specific risk factors

Feature	Effect estimate	Study design *	*n*	References
Female sex	OR 2.21 (95% CI 1.03–4.74)	Multicenter cohort	1479	Moverman 2021[18]
	**OR 1.74**	Multicenter cohort	6320	Moverman 2024 [21]
	OR 2.21 (95% CI 1.03–4.74)	PearlDiver database	67510	Su 2024 [3]
	OR 2.75 (1.45-5.78)	Multicenter cohort	4215	Routman 2020 [38]
Inflammatory arthritis	OR 2.30 (1.02–5.16	Multicenter cohort	1479	Moverman 2021[18]
	RR 3.43	Single-center cohort	335	Moverman 2021[18]
	OR 2.29	Multicenter cohort	6320	Moverman 2024[21]
	OR 3.14 (1.18-6.95)	Multicenter cohort	4125	Routman 2020[38]
	OR 1.99 (1.64-2.41)	PearlDiver database		Su 2024[3]
	OR 4.87 (2.16-10.96)	Single-surgeon cohort	757	Noble 2025[15]
Osteoporosis	OR 2.55 (95% CI 1.24–5.21)	Multicenter cohort	1479	Moverman 2021[18]
	OR 2.00	Multicenter cohort	6320	Moverman 2024[21]
	OR 2.85	Multicenter case-control	2165	Verstraete 2021[19]
	OR 1.98 (1.70-2.31)	PearlDiver database	67510	Su 2024[3]
Rotator cuff disease indication (versus GHOA without cuff pathology)	OR 4.74 (1.84-12.23)	Multicenter cohort	67510	Moverman 2021[18]
	OR 2.36	Multicenter cohort	6320	Moverman 2024[21]
	OR 2.07 (1.24-3.47)	Multicenter cohort	4125	Routman 2020 [38]
	OR 2.79	Multicenter case-control	2165	Verstraete 2021[19]
	OR 1.60 (1.14-2.25)	Multicenter cohort	9079	Roche 2023 [22]
Fracture malunion/nonunion indication	-	Single-center case-control	268	Paszicsnyek 2023[17]
	OR 5.21 (1.20-22.76)	Multicenter cohort	1479	Moverman 2021[18]
Older age	OR 1.02 per 1 year increase in age	Multicenter cohort	6320	Moverman 2024[21]
	-	Multicenter cohort	1479	Moverman 2021[18]
	-	Multicenter cohort	4125	Routman 2020[38]
	-	Single-surgeon cohort	757	Noble 2025[15]
	**OR 1.03**	Multicenter case-control	2165	Verstraete 2021[19]
Smoking	-	Multicenter cohort	1479	Moverman 2021[18]
	-	Multicenter cohort	6320	Moverman 2024[21]
	-	Multicenter cohort	4125	Routman 2020[38]
	-	Single-center cohort	99	Yeazell 2022[23] †
Low acromial BMD (CT-derived)	OR 5.1 (2.4-10.7)	Single-center cohort	263	Colasanti 2025[42] ‡
Acromial morphology	OR 1.14 per 1 mm decrease in acromial thicknes	Single-surgeon cohort	757	Noble 2025[15]
	§	Multicenter case-control	149	Archambault 2024[24]
Prior ipsilateral shoulder surgery	OR 1.82	Multicenter cohort	6320	Moverman 2024 [21]
	OR 2.91 (1.08-7.84)	Multicenter case-control	787	Cho 2021 [2]
Steroid, systemic (chronic)	OR 1.87 (1.58-2.22)	PearlDiver database	67150	Su 2024 [3]
Steroid, intra-articular (within 3 months preceding rTSA)	OR 1.72 (1.36-2.18)	PearlDiver database	67150	Su 2024[3]

* all data are retrospective. ** female patients with inflammatory arthritis subgroup. - not significant. † CT-based volumetric bone density calculation. ‡ direct Hounsfield unit measurement. § regression analysis showed increased scapular spine proportion corresponding to elevated fracture risk. *GHOA* glenohumeral osteoarthritis. *BMD* bone mineral density

Additional interest has been taken in native acromial morphology as a predictor of ASF. A thinner acromion on preoperative CT has been associated with higher fracture rates [15, 23]. A retrospective review of 101 rTSA patients indicated those who developed ASFs had average lateral acromial thickness (in the craniocaudal dimension) of 6.8 mm, compared to patients without fracture with average lateral acromial thickness of 8.7 mm. Posterior acromial thickness was found to be 7.6 mm versus 9.5 mm, respectively [23]. In contrast, findings from a multi-surgeon case-control study suggest the relationship between acromial and scapular spine morphology might better explain fracture risk. Patients with a higher ratio of acromial width (measured in the anteroposterior dimension) compared to medial scapular spine width have been reported to have higher fracture rates [24]. The scapular spine proportion (SSP), defined as the ratio of acromion width to medial scapular spine width, was examined in 51 patients with ASF and 98 without. Those with an SSP of 5 or less demonstrated approximately 5% risk of ASF, whereas patients with an SSP of 9.2 or higher showed 50% risk of fracture [24]. Notably, there was no assessment of the relationship between SSP and fracture zone in this study, so it is not clear the degree to which this effect was driven by an increase in Levy Type III fractures, as might be anticipated with a narrower scapular spine fulcrum relative to a larger acromion lever.

Acromioclavicular joint arthritis with bridging osteophytes has also been associated with higher rates of ASF [25]. The proposed mechanism is osteophyte-mediated load transfer to the base of the acromion. Moreover, acromial geometry may serve to predict fracture pattern in addition to fracture risk. This is hypothesized to be due to differences in deltoid tensioning with variation in acromial slope, as well as different mechanisms of failure at different Levy regions. Previous evidence from a cadaveric model [26] suggests higher acromial slope concentrates strain in the Levy II region as opposed to the Levy III region. Haidamous and colleagues retrospectively reviewed radiographs, finding higher rates of Levy I fractures in higher slope, compared to Levy II fractures in lower slope [27]. These data in combination point to a more anterior point of failure with increased acromial slope. Haidamous et al. also suggest the transverse pattern of many Levy I fractures indicates an avulsion mechanism attributable to the deltoid, whereas the obliquity of most Levy II fractures implies impaction related to impingement of the greater tuberosity on the lateral acromion [27]. These proposed mechanisms identify two different sources of mechanical stress which may be modulated by intraoperative decision-making that considers native acromial morphology.

Intra-articular corticosteroid administration within three months of arthroplasty has also recently been associated with increased risk of ASF [3], despite prior work which failed to demonstrate this relationship while lacking timeline data [3]. While cumulative dose of intra-articular steroid has recently shown association with rTSA revision rate [28], it remains unclear whether the number of preoperative injections impacts ASF risk regardless of temporal relationship to surgery. Systemic steroid use, however, demonstrates a dose-dependent effect. Chronic steroid use is associated with increased ASF risk, whereas patients with a history of single dose of oral steroid within three months of surgery were not at increased risk [3].

### Treatment-Related Risk Factors

RTSA medializes the glenohumeral center of rotation and increases deltoid resting tension and lever arm by increasing the distance between the deltoid origin and insertion. The resulting bending moment applied to the acromion during abduction is significantly greater than in the native shoulder or anatomic total shoulder arthroplasty, predisposing to fatigue failure. Computational models demonstrate these changes concentrate stress in the mid-acromion (Levy zone II), corresponding to the most common fracture location in clinical series [29].

Current rTSA implant designs can be classified into medial glenoid-medial humerus (MGMH), lateral glenoid-medial humerus (LGMH), and medial glenoid-lateral humerus (MGLH) designs [30]. These differ in the location of the center of rotation and the offset of the humerus, and vary in biomechanical features including deltoid efficiency, impingement, and implant loading (Table 2). Roche and colleagues evaluated over 9,000 rTSAs and found design philosophy was associated with ASF risk. MGMH designs demonstrated the highest fracture rate (8.4%) compared to MGLH designs which demonstrated lower rates (2.8%) [22]. However, other contemporary multicenter [21] and comparative [31] studies have suggested LGMH designs may in fact carry higher fracture risk.

**Table 2:** Influence of implant philosophy on acromial biomechanics and fracture risk

Implant Philosophy	Advantages	Disadvantages	ASF implications
Medial Glenoid / Medial Humerus (MGMH) Grammont-style	Medialized COR (at or within 5 mm of joint-implant interface) Increases deltoid moment arm, maximizing efficiency Minimizes glenoid shear, reducing loosening	Greater distalization required for stability Increased cuff tensioning over native state Increased scapular notching	Historically associated with acromial stress fractures because distalization increases deltoid tension[22, 34] Newer multicenter[21] and comparative[33] data suggest **distalization may be less impactful for fracture risk** than excessive glenoid lateralization.
Medial Glenoid / Lateral Humerus (MGLH)	Lateralized humerus reduces scapular notching, improves ER/IR Restores more anatomic contour Preserves reduced torque at glenoid implant-bone interface	Increased deltoid wrapping, reducing force required for deltoid abduction Potential overstuffing/ distalization particularly with onlay stems	**Intermediate fracture risk profile** with some data suggesting slight but insignificant increase in ASF risk[35] and others suggesting no change[34] in fracture risk or even a decrease in acromial stress[21].
Lateral Glenoid / Medial Humerus (LGMH)	Lateralized glenoid reduces scapular notching, improves ER	Increased glenoid shear forces and torque Greater deltoid force requirements	**Strongest association with ASF risk**, with biomechanical data suggesting increased acromial stress[36] and clinical data demonstrating increased fracture risk[33].

*COR* center of rotation,* ER* external rotation, *IR* internal rotation

### Distalization

MGMH (Grammont-type) designs rely on increased distalization to achieve stability and adequate deltoid tensioning for optimal muscular efficiency. Distalization is described as the change in acromiohumeral distance, or delta AHD, between pre- and post-operative states. A finite element analysis performed by Patterson and colleagues identified that humeral distalization with onlay stems increases acromial and scapular spine stress compared to inlay stems which produce less distalization [32]. The ShARC group examined outcomes in 470 rTSA patients, of whom 26 developed ASF, finding each centimeter increase in delta-AHD conferred a 121% increased fracture risk, making distalization the primary modifiable implant-related risk factor in the study [33]. Similarly, Cho et al. identified increased deltoid length as a predictor of ASF risk [2]. They conclude that minimizing distalization is a reasonable intraoperative goal for ASF prevention. Increased distalization may also be the mechanism connecting cuff tear arthropathy to ASF development: because cuff tear arthropathy confers baseline superior migration, typical distalization referenced off the glenoid can be even more excessive compared to patients’ pre-morbid state. However, distalization angle and change in distalization between pre- and postoperative states was not associated with ASF in the recent retrospective review of over 6,000 rTSAs conducted by the ASES multicenter research group [21].

In contrast, inferior glenosphere positioning (inferiorization), reduces acromial bending stress and may help prevent ASF development [29]. However increased inferior glenosphere overhang beyond the native glenoid rim has been associated with increased stress [33]. Ultimately the relationship between inferiorization and ASF risk is difficult to ascertain and the impact of inferiorization is likely mediated by distalization of the entire construct with an inferiorized glenoid.

### Lateralization

The MGMH philosophy has several drawbacks including reduced internal and external rotation, notching, and distalization, which subsequent prosthetic designs attempt to address. Lateralization can occur at the glenoid, humerus, or both, and the origin of lateralization in the construct appears to be relevant to ASF risk. Biomechanical studies suggest increasing glenoid lateralization increases acromial stress [29, 34, 35]. Clinical data, however, is mixed, with some studies indicating glenoid lateralization of 6–8 mm does not independently increase fracture risk [33, 36] and others associating glenoid lateralization with higher risk [21, 31], even in patients with minimal humeral distalization [31]. New evidence demonstrates selective medialization of the glenoid in high-risk patients may mitigate ASF risk [31]. While previous work suggested humeral lateralization may also produce a slight increase in acromial stress [34], more contemporary investigation has failed to support this [21, 37]. As implant designs evolve, further prospective investigations are necessary to assess the influence of glenoid, humeral, and global lateralization on ASFs.

### Other Intraoperative Decision-Making

Glenoid baseplate screw number, placement, and length may also contribute to ASF pathophysiology, driven by the hypothesis that the posterosuperior screw in particular serves as a stress riser. Recently, Routman and colleagues associated ASF with increased number of baseplate screws [38]. Previously, Kennon et al. reported increased risk of ASF with superior screws placed above the central glenoid axis, as well as inferior load to failure in a cadaveric model with the same [39]. This prompted some authors to suggest omission of the posterior screw or selection of a unicortical screw in the superior position to avoid fracture [40].

Aside from implant selection and placement, other intra-operative decisions have been shown to modulate ASF risk. The coracoacromial ligament (CAL) functions as a secondary restraint against superior humeral migration. Transection during rTSA or prior surgery eliminates this load-sharing mechanism, increasing acromial bending strain in a cadaveric model [36] regardless of implant. Preservation of the CAL is therefore biomechanically preferrable and should be considered when feasible, particularly in patients with multiple other ASF risk factors.

### Postoperative Protocol

Finally, there is burgeoning interest in the role of postoperative protocols on ASF development. A recent retrospective database study suggested physical therapy initiation within 6 weeks of rTSA specifically in osteoporotic patients was associated with increased ASF risk [3]. This effect was not seen in patients without osteoporosis. However, the optimal timing and intensity of rehabilitation remain undefined. Critically, the available data cannot establish causality given limitations of the database study design, including inability to assess detailed therapy protocols.

## Prevention

Literature on ASF prevention is largely limited to biomechanical and retrospective studies. Opportunities to intervene include preoperative risk stratification and optimization of bone health, critical evaluation of intraoperative decision-making, and thoughtful management of postoperative rehabilitation.

Preoperative tools such as DEXA or CT-based acromial morphometry have been proposed for systematically identifying patients with increased ASF risk. Patients often arrive to surgical consultations with advanced imaging completed, and CT-based protocols for evaluating acromial characteristics have been derived with excellent inter-rater reliability [41], identifying parameters such as elevated SSP ratios [24] or lower acromial bone mineral density [41, 42], as predictors of ASF. Proposed thresholds corresponding to increased fracture risk include preoperative trabecular bone density of < 99 Hounsfield units (HU) in any of the three Levy zones [42]. While Eisenberg et al. were recently unable to demonstrate a retrospective relationship between anti-resorptive or anabolic therapies and ASF risk [43], no RCT exists describing whether these medications may be useful preoperatively for ASF prevention. They may be considered in high-risk patients for preoperative bone health optimization. Similarly, in patients with known osteoporosis, vitamin D and calcium repletion should be confirmed. No data exists regarding the clinical or cost effectiveness of performing an imaging screening-driven metabolic bone health workup for pre-rTSA patients to reduce postoperative fracture risk. This represents an opportunity for further prospective investigation which may help optimize both patient outcomes and health system workflow.

Patients with inflammatory arthritis are particularly high-risk for ASF [15, 20]. Close coordination with rheumatology to optimize disease activity and disease-modifying anti-rheumatic drugs may prove beneficial in the perioperative period. Given that chronic oral corticosteroid use [3] and corticosteroid injection within three months of surgery [3] have been independently associated with ASF, collaboration to minimize perioperative use of steroids may be beneficial.

Intraoperative decision-making to mitigate ASF risk should consider implant positioning and prioritize preservation of secondary stabilizers. Avoidance of both excessive distalization and glenohumeral impingement is a key surgical goal, as increased acromiohumeral distance has been identified as a modifiable risk factor. While glenoid-sided lateralization up to 8 mm was not independently associated with increased fracture risk in the setting of a 135° inlay humeral component [33], selective medialization of the glenoid in patients with multiple ASF risk factors may be useful in reducing fracture risk [31]. Excessive inferior overhang should be avoided [33]; glenoid component positioning flush with the inferior rim is preferred for mitigating ASF risk. Preservation of the coracoacromial ligament (CAL) should be considered when feasible, as its transection has been shown to increase acromial strain by over 30% [36]. In cases where the CAL has been previously sacrificed, implant choices reducing deltoid loading may be warranted. Finally, adjunctive strategies such as distal clavicle excision have shown preliminary promise in reducing acromial stress through unloading of the acromioclavicular joint, though current evidence remains limited [44]. Collectively, these considerations support a surgical strategy focused on limiting distalization, judicious use of lateralization, and preserving native soft tissue stabilizers. Evidence-based parameters for an optimized rehabilitation protocol have not been defined.

### Treatment

#### Non-Operative Management

ASFs are most commonly managed non-operatively. Sling immobilization, activity restriction, temporary cessation of physical therapy, and serial clinical and radiographic follow-up is employed in 77–88% of ASF cases [45]. It is recommended to initiate sling immobilization as soon as suspicion for ASF arises, even while awaiting additional clinical evaluation or further imaging. Slings are typically maintained for six to eight weeks, with CT reassessment recommended if clinical improvement plateaus or radiographic displacement progresses. Off-label intranasal calcitonin is being investigated as an adjunct to immobilization for non-displaced fractures or early stress reactions, with the hypothesis that its antiresorptive effect counters the repetitive stress across a compromised area of bone and its analgesic effects relieve symptoms that contribute to diminished function [46]. As investigation remains in early feasibility stages, intranasal calcitonin’s impact on the natural history of ASRs and nondisplaced ASFs represents a promising area of future study.

Despite remaining the standard of care, conservative management produces significantly worse functional outcomes across all patient-reported outcome measures compared to non-fracture rTSA controls [4]. Outcomes are location-dependent: Levy Type I and IIA fractures achieve outcomes approaching non-fracture rTSA, while Types IIB, IIC, and III demonstrate worsened function, progressive scapular tilt, and osteolysis [4, 9]. Patients with Type III fractures demonstrate no functional improvement from their pre-rTSA baseline [9].

Radiographic nonunion may occur in over 60% of conservatively managed patients, with a 27.8% dissatisfaction rate [9]. Yet fracture consolidation does not translate to improved clinical outcomes compared to persistent nonunion [1], suggesting the biomechanical insult of the fracture event may drive long-term impairment, highlighting the value of prevention.

Fracture displacement is an independent prognostic variable. Haidamous et al. reported only 55% of nonoperatively-treated ASF patients achieved the minimal clinically important difference (MCID) for improvement in ASES score at two-year follow-up, and fracture displacement was a more powerful predictor of failure to achieve MCID than Levy type [27]. This support efforts to achieve shorter time to diagnosis, before displacement occurs, and a lower threshold for CT imaging in symptomatic patients when plain radiographs appear unremarkable. The clinical value of early intervention to prevent displacement has not been independently assessed and represents an important area of future study.

Nonoperatively-managed ASF patients demonstrate lower subsequent revision arthroplasty rate (3.2%) compared to operatively treated patients (7.0%), although both groups demonstrate increased need for revision compared to patients without fracture (2.3%) [3]. These findings favor nonoperative management as an appropriate initial strategy for many fractures. However, it must be recognized that this finding may be confounded by the more severe ASF features that are understood as indications for primary operative intervention, making those who are treated operatively a more complex subpopulation potentially at elevated risk of revision regardless of management.

### Operative Management

**Fig. 3:**
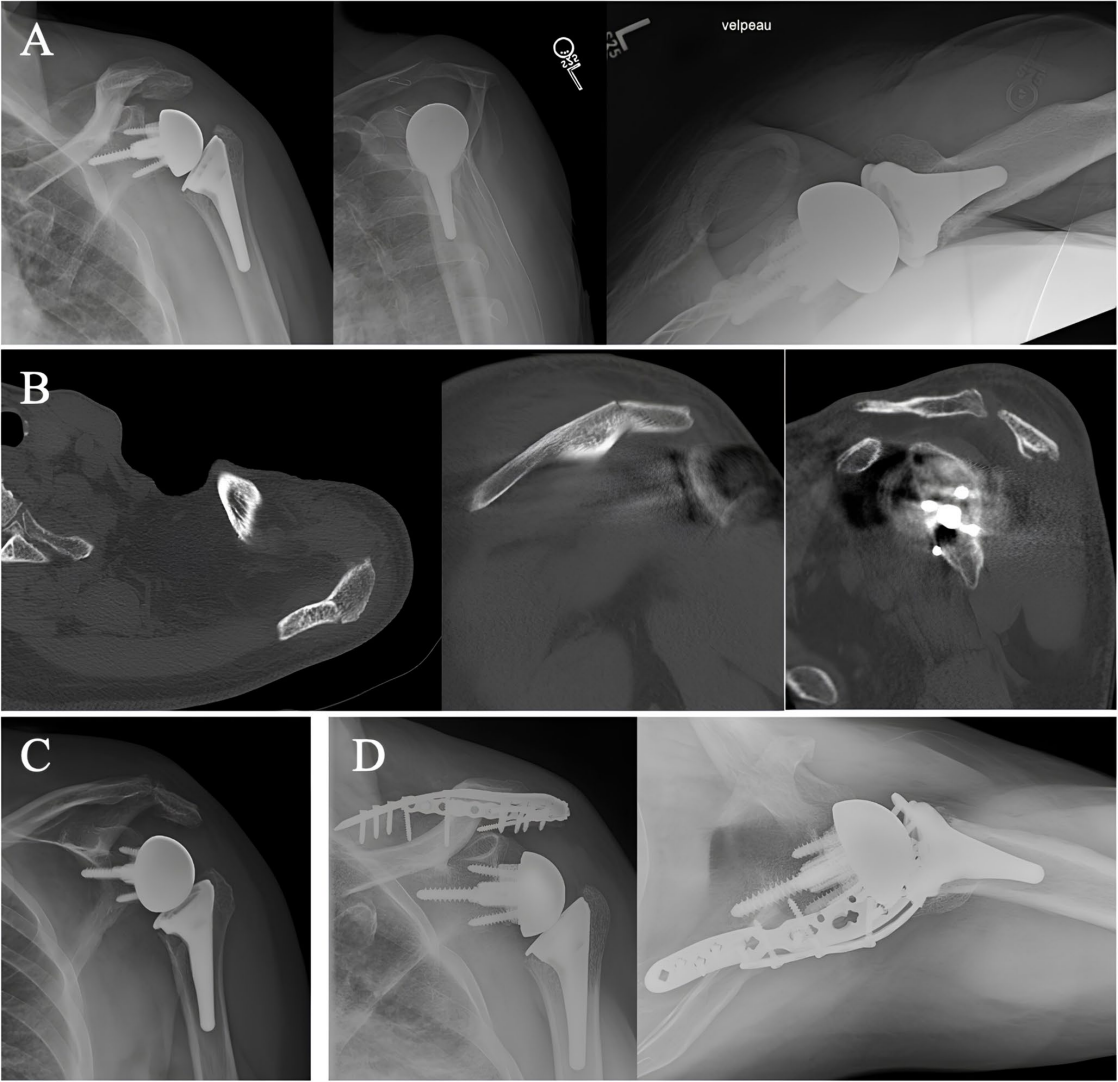
66-year-old male with acute shoulder pain three months after primary rTSA, with a nondisplaced Levy zone IIB acromial stress fracture (**A**, **B**) which displaced with nonoperative management (**C**) and was successfully managed with dual plating (**D**).

Surgical fixation is generally considered for displaced fractures (particularly Levy Types IIB through III) with scapular tilt and compromised deltoid function, progressive displacement during conservative management (Fig. 3), and established symptomatic nonunion with functional deficits. It is important to note that no RCTs exist comparing operative to nonoperative management. In the largest comparative study to date, Schenk et al. found no statistically significant difference in Constant Score, Shoulder Subjective Value (SSV), or range of motion between operatively and non-operatively treated groups, with both performing worse than fracture-free rTSA controls [7]. Operative treatment has been found to achieve higher radiographic union rates, however as discussed this has not consistently corresponded to improved clinical outcomes [1]. Current indications for surgery are based largely on expert consensus.

Operative management of persistently symptomatic Levy type I fractures may consist of fragment excision or fixation. Excision can be accomplished open, or arthroscopically as in the technique described by Hartzler and colleagues [47]. When operative management is selected for Levy types II-III, ORIF with plate-and-screw constructs is the most common technique. Cassidy et al. examined fixation across 18 fractures found single-plate fixation most common for Levy Type II fractures, while dual plating was preferred for Type III fractures, aiming to provide rotational stability against the multi-directional distraction forces of the deltoid insertion [48]. Radiographic union was achieved in 83.3% of operatively-treated patients. Tension band fixation has also been reported however is largely limited to older small case reports; contemporary reports support plating constructs.

No study has comparatively evaluated the impact of bone grafting at the time of ORIF for ASFs. Hagelstein and colleagues reported a series of 25 ASFs treated with dual plating and autologous iliac crest bone grafting, reporting significant improvements in active range of motion and a 57% radiographic union rate [49]. However, 23% of patients experienced donor site fracture, which can confer significant morbidity in this elderly population [49]. Other series do not directly report on the presence of bone graft or its origin in their constructs [1, 9, 48], precluding evaluation of the utility of bone grafting over internal fixation alone. The literature also lacks data pertaining to the use of biologic augmentation such as bone morphogenetic protein (BMP) or demineralized bone matrix (DBM) in the specific context of ASFs. This represents an important area of future study. In our practice, if patients have osteoporotic bone, we do everything possible to promote the most durable construct while also adding biologic augments. A structural fibula allograft is placed across the fracture site and commercially available biologic demineralized bone matrix (DBM) is placed at the fracture site while compressing the fracture using Arbeitsgemeinschaft für Osteosynthesefragen (AO) principles.

Additionally, no group has published on rTSA revision for the indication of ASF. While the biomechanical rationale for implant repositioning is compelling [21, 31, 35], several factors have likely limited investigation of this approach. This complication is relatively uncommon, and patients who sustain ASFs often bear substantial medical comorbidities. Expected functional gains from prosthetic revision may be modest relative to the risk profile of revision surgery. While the overall biomechanical premise is attractive, the absence of clinical evidence and the considerable morbidity associated with prosthetic revision limit current enthusiasm for this as a strategy to treat ASF.

Overall, classification-driven treatment provides a clinically intuitive framework, though evidence supporting a formal algorithm for ASF remains limited. To preserve deltoid attachment integrity, Levy Types I and IIA fractures are generally managed conservatively, given their favorable prognosis [9]. Types IIB and IIC warrant initial conservative management with operative escalation for progressive displacement or failure to improve. Type III scapular spine fractures present the strongest rationale for surgical consideration given near-universal failure to achieve MCID for functional improvement with conservative management [9, 10].

## Conclusion

ASF represents a persistently difficult rTSA complication, impairing postoperative function and seeing limited improvement with current treatment options. Understanding patient- and treatment-related contributors can help surgeons mitigate risk and aid in perioperative counseling and management. Areas for future study include development of cost-effective screening criteria to identify high-risk patients, and optimization of preoperative metabolic bone health interventions, including more robust characterization of the role of intra-articular steroid in ASF development. Additional prospective data are necessary to further adjudicate the influence of implant positioning and design parameters to better inform implant development. Early intervention for stress reactions and nondisplaced fractures warrant further investigation, particularly calcitonin’s potential role in pain control and ASF prevention.

## Key References

- Levy JC, Anderson C, Samson A. Classification of postoperative acromial fractures following reverse shoulder arthroplasty. *J Bone Joint Surg Am.* 2013;95(15). doi:10.2106/JBJS.K.01516. Introduced the Levy classification system for acromial stress fractures after rTSA.
- Su F, Kucirek N, Goldberg D, Feeley BT, Ma CB, Lansdown DA. Incidence, risk factors, and complications of acromial stress fractures after reverse total shoulder arthroplasty. J Shoulder Elbow Surg. 2024;33(1):65–72. doi: 10.1016/j.jse.2023.06.008. Identified osteoporosis, rheumatologic disease, chronic oral corticosteroid use, and preoperative intra-articular corticosteroid injection within 3 months of surgery as independent risk factors for ASF.
- Moverman MA, Puzzitiello RN, Glass EA, Swanson DP, Efremov K, Lohre R, et al. Implant-Positioning and Patient Factors Associated with Acromial and Scapular Spine Fractures After Reverse Shoulder Arthroplasty: A Study by the ASES Complications of RSA Multicenter Research Group. *J Bone Joint Surg Am*. 2024;106(15):1384–94. doi: 10.2106/JBJS.23.01203. Identified female sex, osteoporosis, and increased distalization as factors increasing stress on the acromion and scapular spine.
- Roche CP, Fan W, Simovitch R, Wright T, Flurin PH, Zuckerman JD, et al. Impact of accumulating risk factors on the acromial and scapular fracture rate after reverse total shoulder arthroplasty with a medialized glenoid-lateralized humerus onlay prosthesis. J Shoulder Elbow Surg. 2023;32(7):1465–75. doi: 10.1016/j.jse.2022.12.026. Demonstrated cumulative impact of multiple risk factors, including osteoporosis, rheumatoid arthritis, female sex, and greater arm lengthening and deltoid tension on ASF risk. *J Shoulder Elbow Surg.* 2023;32(7):1465–1475. doi:10.1016/j.jse.2022.12.026.
- Eisenberg MT, Hui C, Schwartz K, Qubain L, Amini MH**.** In reverse shoulder arthroplasty, selectively medializing the glenoid in higher-risk patients is associated with reduced risk of acromial stress fracture. *J Shoulder Elbow Surg.* 2026. doi:10.1016/j.jse.2026.04.029. Demonstrated that selective glenoid medialization may reduce ASF incidence in high-risk patients, suggesting a modifiable surgical factor.
- Mandalia K, Gulotta L, Ross G, Shah S. Safety and Early Results for Off-Label Use of Intranasal Calcitonin for Treatment of Nondisplaced Acromial and Scapular Spine Stress Fractures After Reverse Total Shoulder Arthroplasty. J Am Acad Orthop Surg Glob Res Rev. 2024;8(4). doi: 10.5435/JAAOSGlobal-D-24-00045. Reported favorable early outcomes with off-label intranasal calcitonin as an adjunct for treating nondisplaced acromial and scapular spine stress fractures, suggesting a potential role in fracture healing and symptom management. *J Am Acad Orthop Surg Glob Res Rev.* 2024;8(4). doi:10.5435/JAAOSGlobal-D-24-00045.

## Data Availability

No datasets were generated or analysed during the current study.
